# Identification of stable reference genes for qPCR studies in common wheat (*Triticum aestivum* L.) seedlings under short-term drought stress

**DOI:** 10.1186/s13007-020-00601-9

**Published:** 2020-04-25

**Authors:** Karolina Dudziak, Magdalena Sozoniuk, Hubert Szczerba, Adam Kuzdraliński, Krzysztof Kowalczyk, Andreas Börner, Michał Nowak

**Affiliations:** 1 Institute of Plant Genetics, Breeding and Biotechnology, University of Life Sciences in Lublin, Akademicka 15, 20-950 Lublin, Poland; 2 Chair and Department of Biochemistry and Molecular Biology, Medical University of Lublin, Chodźki 1, 20-093 Lublin, Poland; 3 Department of Biotechnology, Microbiology and Human Nutrition, University of Life Sciences in Lublin, Skromna 8, 20-704 Lublin, Poland; 4 Leibniz Institute of Plant Genetics and Crop Plant Research (IPK), Corrensstrasse 3, Stadt Seeland, 06466 Gatersleben, Germany

**Keywords:** Reference genes, Drought, Osmotic stress, Common wheat, qPCR, In silico analysis

## Abstract

**Background:**

Quantitative PCR (qPCR) is one of the most common and accurate methods of gene expression analysis. However, the biggest challenge for this kind of examinations is normalization of the results, which requires the application of dependable internal controls. The selection of appropriate reference genes (RGs) is one of the most crucial points in qPCR data analysis and for correct assessment of gene expression. Because of the fact that many reports indicate that the expression profiles of typically used RGs can be unstable in certain experimental conditions, species or tissues, reference genes with stable expression levels should be selected individually for each experiment. In this study, we analysed a set of ten candidate RGs for wheat seedlings under short-term drought stress. Our tests included five ‘traditional’ RGs (GAPDH, ACT, UBI, TUB, and TEF1) and five novel genes developed by the RefGenes tool from the Genevestigator database.

**Results:**

Expression stability was assessed using five different algorithms: geNorm, NormFinder, BestKeeper, RefFinder and the delta Ct method. In the final ranking, we identified three genes: CJ705892, ACT, and UBI, as the best candidates for housekeeping genes. However, our data indicated a slight variation between the different algorithms that were used. We revealed that the novel gene CJ705892, obtained by means of in silico analysis, showed the most stable expression in the experimental tissue and condition.

**Conclusions:**

Our results support the statement, that novel genes selected for certain experimental conditions have a more stable level of expression in comparison to routinely applied RGs, like genes encoding actin, tubulin or GAPDH. Selected CJ705892 gene can be used as a housekeeping gene in the expression analysis in wheat seedlings under short-term drought. The results of our study will be useful for subsequent analyses of gene expression in wheat tissues subjected to drought.

## Background

Quantitative PCR (qPCR, real-time PCR) is a widely applied method in the analysis of gene expression due to its high sensitivity, high specificity and good reproducibility [1–3]. However, for proper analysis of gene expression involving qPCR, a normalization step is necessary. The most common strategy is based on reference genes (RGs), also called ‘housekeeping genes’, which are internal controls with stable expression levels in the tested material under the experimental conditions. Therefore, the selection of appropriate RGs is one of the most crucial points in qPCR data analysis and for correct assessment of gene expression [4, 5]. Numerous housekeeping genes, such as actin (ACT), tubulin (TUB), and 18S ribosomal RNA (18S rRNA), that are necessary for proper cellular metabolism are widely used as RGs in many studies. Nevertheless, many reports indicate that the expression profiles of these genes can be unstable in certain experimental conditions, species or tissues [1, 4, 6]. Many authors suggest that there is no universal RG for all experimental subjects [e.g. 2, 6–9]. Each experiment requires the selection of an ideal RG [10]. Many studies have been conducted on the selection of appropriate RGs for *Arabidopsis thaliana* [11], soya [12], peach [13], rice [14], cotton [15, 16], and poplar [1]. However, there are still few studies concerning wheat [2, 3, 17], especially in response to abiotic stresses such as drought. There are data for wheat infected by *Puccinia* spp. [18], by BYDV-PAV and BYDV-PAS viruses [19] or under different farming conditions (nitrogen fertilization and type of system) [2]. For rice in water shortage conditions, the gene encoding ubiquitin was identified as a RG [20]. However, the selection of RG for gene expression analysis under drought conditions in wheat remains a major challenge. Most of the reports concerning RG tests have been focused on validating a set of commonly used reference genes. Currently, many studies show that the identification of ideal RGs can be based on in silico analysis, such as the Genevestigator database and the RefGenes tool [21]. The Genevestigator database contains a large set of systematically annotated and quality-controlled microarray data from several organisms [22], and RefGenes is an online tool that utilizes this database to enable users to search for genes that exhibit minimal expression variance across a chosen set of arrays. This method ensures the identification of genes with more stable expression than the standard genes [1, 2, 16, 23, 24]. For the analysis of the results and selection of the best RG, numerous platforms using different algorithms have been developed, including geNorm [25], NormFinder [26], BestKeeper [27] and RefFinder [28].

In this study, we conducted an analysis using geNorm, NormFinder, BestKeeper and RefFinder to select the most suitable RG for wheat plants in the seedling stage when tested under drought conditions. Moreover, a method of directly delta Ct analysis based on comparisons between each RG and the other RGs within each sample and calculation the average standard deviation against the other RGs [29] was performed.

## Results

### Selection of candidate reference genes using the RefGenes tool

RefGenes is an in silico method enabling the identification of genes with high expression stability within microarray libraries of wheat subjected to drought. Using this tool to examine normalized and well-annotated microarray experiments, we found 20 candidate RGs. Among these genes, we selected five candidates with stable expression levels under drought conditions. The candidate RGs obtained in this analysis were used for validation in qPCR (Table 1).

**Table 1:** Primers sequences and amplicons characteristics of candidate RGs

Gene name	GenBank accession number	Primer sequence (5′ → 3′)	Amplicon length (bp)	Reference
*Triticum aestivum* alpha-tubulin mRNA (*TUB*)	U76558	F: CCCTGAGGTTTGATGGTGCT	156	Rampino et al. [40]
		R: TGGTGATCTCAGCAACGGAC		
*Triticum aestivum* mRNA for actin (*ACT*)	AB181991	F: GGAGAAGCTCGCTTACGTG	136	Wei et al. [43]
		R: GGGCACCTGAACCTTTCTGA		
*Triticum aestivum* glyceraldehyde-3-phosphate dehydrogenase (GAPC) mRNA (*GAPDH*)	EF592180	F: AACGACCCCTTCATCACCAC	150	Wei et al. [43]
		R: GTTCCTGCAGCCAAACACAG		
*Triticum aestivum* ubiquitin (WUB1) mRNA (*UBI*)	AY297059	F: GGAGTCCACCCTTCACTTGG	130	Li et al. [41]
		R: GACACAGGCACCATTCGAG		
Wheat translation elongation factor 1 alpha-subunit (TEF1) mRNA (*TEF1*)	M90077.1	F: AGGCTGACTGTGCTGTTCTC	106	Liu et al. [42]
		R: AGAGTGAAAGCAAGGA		
EST BJ254354	BJ254354	F: TGTTGAGGAGACAGTTGCCC	101	This study
		R: GTTTGTCGGGCAAfTGCAGAG		
EST wpa1c.pk012.d13	CA596223	F: AGAACTTGGCGTACAGGCTC	109	This study
		R: GGCAGAGACTCGTACATCGG		
EST wdi1c.pk002.n12	CA728440	F: CCCATCCAGCTCACACTGAC	134	This study
		R: CGTGTCCGGCTTAAAACGAG		
EST CJ705892	CJ705892	F: GCCTCAGTGGTAGGAGCATT	116	This study
		R: TTCAGCAAATGCGGTGGTTG		
EST wre1n.pk0067.d7	CA644093	F: CAGTCTGCACTGTGGCACTA	113	This study
		R: CCAGCCGCCTAAACTTCTGA		

### Expression levels of the reference genes

**Fig. 1:**
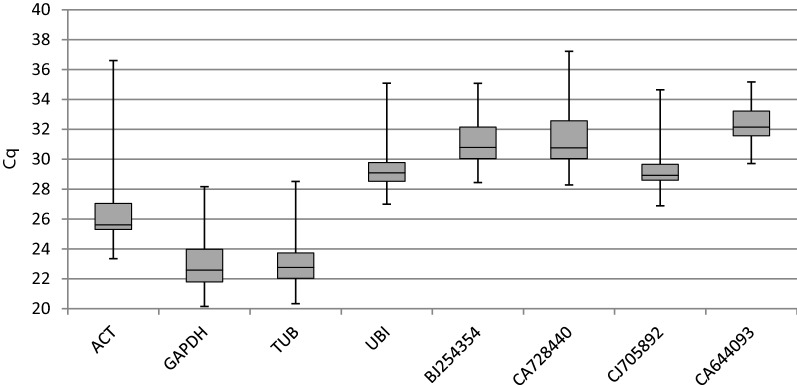
Cq values for eight candidate reference genes across experimental samples. A line across the box is depicted as the median. The box indicates the 25th and 75th percentiles, the whiskers represent the maximum and minimum values

To identify the most stable housekeeping genes, cDNA of all tested lines (stress imposed and control) was used in qPCR. The specificity of the primers was estimated by qPCR melting curve analysis. A single peak of the melting curve was observed for most of the tested primers (8 of 10 primer pairs), confirming the specificity of the amplicons. Only for two primer pairs (for the TEF1 and CA596223.1 genes) unspecific products of expression were observed, and because of that fact, they were excluded from further analysis. Moreover, no signal was detected in the NTC samples. We used the standard curve method with a pool of all the cDNAs to determine the PCR efficiency (E) and the correlation coefficient (R^2^) for each primer pair. The obtained results were analysed according to Bustin et al. [10], and the results indicated that the acceptable range of efficiency was from 80 to 120%. According to Tyburski et al. [30], a slope equal to − 3.32 is evidence of high reaction efficiency, and R^2^ = 1 indicates that the same expression level was observed in the calibrator and tested sample. We obtained E values varying between 83.01% and 112.75% and R^2^ from 0.83 to 1 (Table 2). The raw quantification cycle (Cq) values were estimated for determination of the gene expression levels. The Cq values for analysed samples ranged between 20.16 and 37.60 (Fig. 1).

**Table 2:** Slope, efficiency and R^2^ values for analyzed candidate RGs

Gene	Slope	Efficiency [%]	R^2^
*ACT*	− 3.32	100.00	1
*GAPDH*	− 3.36	98.44	1
*TUB*	− 3.41	96.45	1
*UBI*	− 3.23	103.98	0.99
BJ254354	− 3.53	91.99	0.93
CA728440	− 3.81	83.01	0.94
CJ705892	− 3.25	103.09	0.98
CA644093	− 3.05	112.75	0.83

### Expression stability of the reference genes

The expression stability of selected RGs was estimated using five different algorithms: geNorm, NormFinder, BestKeeper, RefFinder and the delta Ct method. For each platform, eight RGs were ranked from the most stable to the least stable. In the results generated by the software packages, differences were observed. The ranking of RGs using geNorm was mostly in agreement with that of NormFinder. We found that the first three genes with the most stable expression and the gene with the least stable expression were the same for these two platforms. RefFinder and delta Ct analysis gave the same rankings among all 8 RGs. However, the results of BestKeeper and RefFinder showed different rankings for the most and least stable candidate genes (Table 3).

**Table 3:** geNorm M and stability values (SV) of the eight candidate reference genes obtained by geNorm, NormFinder, BestKeeper, RefFinder algorithm and delta Ct method

Rank	geNorm		NormFinder		BestKeeper		RefFinder		Delta Ct	
	Gene	geNorm M	Gene	SV	Gene	SV	Gene	SV	Gene	SV
1	CJ705892	0.554	CJ705892	0.072	ACT	0.498	ACT	1.00	ACT	0.87
2	ACT	0.573	ACT	0.084	CJ705892	0.526	UBI	1.86	UBI	0.89
3	UBI	0.596	UBI	0.131	UBI	0.564	CJ705892	2.71	CJ705892	0.89
4	GAPDH	0.676	TUB	0.139	TUB	0.732	GAPDH	4.43	GAPDH	0.99
5	TUB	0.729	BJ254354	0.152	CA644093	0.761	TUB	4.95	TUB	1.03
6	BJ254354	0.771	GAPDH	0.153	GAPDH	0.842	BJ254354	5.96	BJ254354	1.04
7	CA644093	0.839	CA644093	0.216	BJ254354	0.851	CA644093	6.44	CA644093	1.14
8	CA728440	0.975	CA728440	0.300	CA728440	1.032	CA728440	8.00	CA728440	1.46

### geNorm analysis

geNorm analysis indicated that the stability of gene expression (M-value) varied between 0.550 for the most stable gene and 0.975 for the least stable gene (Table 3). According to this algorithm, genes with the lowest M-value were considered to be the most stable, whereas genes with the highest M-value were considered to be the least stable [31]. Based on geNorm software results, we identified a CJ705892 gene as the most stable in the tested wheat lines. Among a set of commonly used housekeeping genes, actin (0.575) and ubiquitin (0.600) were assessed as the most stable. The rest of the genes obtained from the Genevestigator database indicated a low level of expression stability. The least stable was CA728440 (0.975).

### NormFinder analysis

The stability of the eight selected RGs was further analysed using the NormFinder platform. The NormFinder software analyses datasets and estimates stability based on intra-group and inter-group variation. The genes with lower stability values were considered to be the most stable RGs, whereas the genes with higher stability values were ranked as the least stable [32]. Based on the NormFinder algorithm, we found that the CJ705892 gene (stability value: 0.072) was the most stable gene, followed by ACT (0.084) and UBI (0.131). The least stable was CA728440 (0.300) (Table 3). We observed that the results of NormFinder and geNorm were slightly different. However, both algorithms indicated that the CJ705892 gene and CA728440 were the most and least stable genes, respectively. Therefore, based on the geNorm and NormFinder analysis and previous data concerning the BestKeeper and RefFinder platforms, we concluded that a novel gene, CJ705892, developed by the RefGenes tool, was found to be the most stable RG in tested wheat lines under short-term drought.

### BestKeeper analysis

The obtained data were also analysed using the BestKeeper algorithm. BestKeeper software is usually employed by assessing the correlation coefficients of each individual gene with the geometric mean of all genes (the BestKeeper Index) [33]. These results were different from those of NormFinder and geNorm. According to BestKeeper, the most stable gene was ACT (0.468), followed by CJ705892 (0.526). The least stable was CA728440 (1.032) (Table 3).

### RefFinder analysis

The RefFinder platform requires only raw Cq values without any option to include PCR efficiency. The ranking obtained by this algorithm is based on the standard deviations of the RG Cq values [33]. The results obtained by geNorm and NormFinder were not provided by the RefFinder output. RefFinder assessed ACT (1.00) and UBI (1.861) as the most stable genes. The gene CJ705892 (2.711) was in third place (Table 3).

### Delta Ct method

The results obtained by the delta Ct method showed the same results as we observed using the RefFinder software. The most stable genes were ACT (0.868) and UBI (0.887), followed by CJ705892 (0.895) (Table 3). Analysis of all datasets suggests that the results obtained by the NormFinder, geNorm and BestKeeper methods indicated that the gene CJ705892 is on top of the RG rankings, with some slight differences in the rankings. All statistical algorithms showed CA728440 as the least stable gene.

## Discussion

Analysis of gene expression patterns is the basis for the development of knowledge about the mechanisms involved in the initial reaction of plants to stress. The most accurate technique for expression analysis is qPCR, and a selection of the best RG is a crucial step to avoid experimental errors and incorrect interpretation of results. The ideal internal control has stable expression in the tested material under experimental conditions. In the present study, we analysed 10 potential RGs for wheat substitution lines under short-term drought conditions. We combined four algorithms (RefFinder, BestKeeper, geNorm, and NormFinder) and the delta Ct method to estimate the best RG. In the final ranking, we identified three genes, CJ705892, ACT, and UBI, as the best candidates. However, our data indicated a slight variation between the different algorithms that were used. According to the geNorm and NormFinder platforms, the CJ705892 gene had the most stable expression, while the BestKeeper and RefFinder programs showed this gene in second and third place, respectively. The obtained results are confirmed by numerous studies suggesting that variation is caused by the use of different algorithms [9, 33].

As suggested by previous studies, the most reliable tools for RG stability estimation are geNorm and NormFinder [33]. Many reports based only on these two algorithms have been used for the identification of RGs, e.g., in berry [34], rice [14, 35], tomato [36], soy [37], cotton [16] or wheat [17, 23, 38]. Based on these data, the results generated by geNorm and NormFinder were crucial for RG selection in our study.

The data obtained by BestKeeper demonstrated slight differences compared to those from geNorm and NormFinder. As the best RG, BestKeeper showed actin, followed by the CJ705892 gene and UBI. Thus, the ranking order was not identical; however, the three first genes, which were considered the most stable under the given experimental conditions, were the same. A similar variance was described for apple under postharvest conditions [39], *Caragana intermedia* under osmotic stress [8] and *Actinidia deliciosa* infected by *Pseudomonas syringae* pv. *actinidiae* [9]. Based on previous studies, we suggest that these variances are caused by the BestKeeper algorithm, which employs correlation analyses between the candidate gene Cq and an index derived from the candidate geometric mean. In contrast, the algorithms of the geNorm and NormFinder platforms use variation measures to calculate the stability of gene transcription [39].

The most significant differences were observed using RefFinder software. This program indicated ACT and UBI as the most stable genes, followed by CJ705892. De Spiegelaere et al. [33] investigated differences between all four algorithms that were used in our study. The authors explained that RefFinder ranking is based on the standard deviations of the RG Cq values and that the analysis requires only non-corrected raw Cq values. Moreover, De Spiegelaere et al. [33] suggested that the RefFinder system is applied in many studies of RG validation because it is free and performs a quick analysis using the three most popular algorithms. However, this platform has not been well validated yet and has no scientific basis. Thus, we suggest that RefFinder software should be used as a complementary tool in the analysis of RG stability.

Among the ‘traditional’ RGs, we found that ACT had the most stable expression level. This result was obtained by three software packages: geNorm, NormFinder, and BestKeeper. Our results confirmed a previous study conducted on Chinese Spring wheat treated with different abiotic (nutrient deprivation, hormone application) and biotic (rust infection) stress factors. This analysis performed with the geNorm, NormFinder and BestKeeper packages also showed actin as the best RG.

Our results indicated that a novel gene obtained using the RefGenes tool from the Genevestigator database was the most stable among all the tested genes. Many studies have demonstrated that novel genes selected for experimental conditions have a more stable level of expression. Marcolino-Gomes et al. [24] analysed a number of genes, including genes widely used as references (GAPDH, TUB, β-actin, etc.) and additional genes developed with Genevestigator and RNA-seq libraries. The authors found that some novel genes obtained by in silico analysis indicated stable expression profiles in soy under drought. Similar observations were described for *Triticum aestivum* in different tissues under temperature stress.

Despite slight differences between the rankings obtained by the four different programs, the results of geNorm and NormFinder overlapped and showed CJ705892 as the best RG. Based on previous studies suggesting that these two algorithms are the most reliable, we suggest that CJ705892 can be used as a housekeeping gene in the expression analysis of wheat seedlings under short-term drought. The results of our study provide new information that will be useful in molecular studies of wheat response to water deficiency.

## Conclusions

In our study, we combined analysis based on standard RG and novel genes obtained via RefGenes tool from Genevestigator database in order to identify the optimal RG for common wheat (*Triticum aestivum* L.) seedlings subjected to short-time osmotic stress. qPCR results were analyzed using four different algorithms. Our study allowed for the identification of the novel gene showed the most stable expression level in tested lines of *Triticum aestivum* L. under water deficit, which can be used as RG for subsequent experiments based on similar plant material and conditions–wheat seedlings subjected to water deficit stress.

## Methods

### Plant materials and stress induction

In our study, the set of 18 inter-varietal single chromosome substitution lines (ISCSLs) of *Triticum aestivum* L. were used. ISCSLs based on the drought-tolerant cultivar ‘Saratovskaya 29’ (S29) as a recipient and the drought-sensitive cultivar ‘Janetzkis Probat’ (JP) as a donor were used in the study.

For the induction of germination, sterilized kernels were incubated at 4 °C for 48 h. Then, the kernels were germinated in Petri dishes containing filter papers soaked in distilled water in the dark at 24 °C. After 2 days, seedlings were transferred into plastic pots containing full-strength Murashige Skoog (MS) medium. Plants were grown under controlled conditions in a hydroponic culture in a phytotron greenhouse for 5 days under control conditions (light/dark regime of 16/8 h at 25 ± 3 °C, relative humidity of 50 ± 10%, and the light intensity during the day time was 350 μmol m^−2^ s^−1^). Seven-day-old seedlings were treated with 10% polyethylene glycol (PEG-6000) dissolved in MS solution to induce drought stress. Seedlings without roots were collected after 1, 3 and 6 h of stress treatment. Plants growing in MS medium without PEG were used as a control.

### Total RNA isolation

RNA extraction was performed after 0, 1, 3 and 6 h of exposure to stress. After harvesting, plant material was immediately frozen in liquid nitrogen and grind to a fine powder with mortar and pestle. Total RNA was isolated using TRIzol reagent (Invitrogen) according to the manufacturer’s instructions. The quality and quantity of RNA samples were assessed on 2% agarose gel electrophoresis and spectrophotometrically using the DeNovix DS-11 (DeNovix).

### Reverse transcription

Reverse transcription PCR was performed with an iScript™ cDNA Synthesis Kit (Bio-Rad) following the manufacturer’s instructions. RT-PCRs were carried out in a total volume of 30 µl containing 1.5 µg of the total RNA, 6 μl 5× iScript Reaction Mix containing blend of oligo(dT) and random hexamer primers and 1.5 μl of iScript Reverse Transcriptase, which is modified Moloney murine leukemia virus (MMLV) reverse transcriptase. The thermal conditions applied were as follows: priming for 5 min at 25 °C; reverse transcription for 20 min at 46 °C and inactivation for 1 min at 95 °C. Obtained cDNA was stored in -25 °C.

### Selection of candidate reference genes

To identify the gene with the most stable expression in common wheat under drought treatment, a set of ten genes were selected and tested. Five genes commonly used as internal controls in wheat were obtained from previous expression studies and included TUB, ACT, GAPDH, UBI and TEF1 [40–43]. Five novel genes were identified as potential references via the RefGenes in silico tool from the Genevestigator platform [https://www.genevestigator.com/gv/plant.jsp] [21] (Table 1). The Genevestigator database provides normalized and well-annotated microarray tests. The RefGenes tool enables searching for genes with minimal expression variance across a chosen set of arrays on the Genevestigator platform [22, 24].

### Design of qPCR primers and amplification efficiency testing

The sequences of all tested gene transcripts were obtained from the NCBI database. Primers for qPCR were designed using the Primer-BLAST tool [44] (Table 1). The same tool was used for determination of the primer’s specificity in silico. The PCR amplification efficiency was determined for each primer pair by the analysis of the slope obtained from a standard curve generated from a serial dilution of pooled cDNA as reported previously [32]. The amplification efficiency (E) and correlation coefficient (R^2^) of the primers were calculated according to the equation [10^(1/−S)−^1] × 100%, where S represents the slope of the linear regression.

### Quantitative PCR (qPCR) conditions

Quantitative PCR (qPCR) was performed based on SYBR Select Master Mix (Applied Biosystems) according to the manufacturer’s instructions. PCRs were carried out in a total volume of 20 µl containing 800 ng of cDNA, 1 × SYBR Select Master Mix (Applied Biosystems) based on AmpliTaq^®^ Fast DNA Polymerase and 400 nM of each primer. qPCR was performed on a LightCycler^®^ 96 System (Roche) under the following thermal conditions: 2 min at 50 °C; 10 min at 95 °C; 40 cycles of 15 s at 95 °C and 1 min at 60 °C. Each reaction was carried out in three technical replicates along with a no template control (NTC). To confirm the amplification specificity and lack of primer dimer formation, each run was performed with a melting curve analysis. Each sample was analyzed in two full biological and three technical replications at the qPCR level.

### Analysis of gene expression stability

The raw data of qPCR was processed by means of LightCycler^®^ 96 software v. 1.1 (Roche). The expression stability of the ten selected housekeeping genes (TUB, ACT, GAPDH, UBI, TEF1, BJ254354, CA596223, CA728440, CJ705892, and CA644093) in wheat seedlings under drought conditions were analysed using the RefFinder [28], geNorm [25], BestKeeper [27] and NormFinder [26] software packages and the delta Ct (dCt) method [29]. Raw Cq values were used in the BestKeeper and delta Ct algorithms. For the geNorm and NormFinder analysis, raw Cq values were transformed into relative quantities.

## Data Availability

All data generated or analysed during this study are included in this published article and its supplementary information file.
